# Rheology of Gels and Yielding Liquids

**DOI:** 10.3390/gels9090715

**Published:** 2023-09-03

**Authors:** Alexander Ya. Malkin, Svetlana R. Derkach, Valery G. Kulichikhin

**Affiliations:** 1 A.V. Topchiev Institute of Petrochemical Synthesis, Russian Academy of Sciences, Leninskii Prosp. 29, 119991 Moscow, Russia; klch@ips.ac.ru; 2 Laboratory of Chemistry and Technology of Marine Bioresources, Institute of Natural Science and Technology, Murmansk State Technical University, 183010 Murmansk, Russia; derkachsr@mstu.edu.ru

**Keywords:** gel, gel-like state, yield stress, yielding liquids, flow

## Abstract

In this review, today’s state of the art in the rheology of gels and transition through the yield stress of yielding liquids is discussed. Gels are understood as soft viscoelastic multicomponent solids that are in the incomplete phase separation state, which, under the action of external mechanical forces, do not transit into a fluid state but rupture like any solid material. Gels can “melt” (again, like any solids) due to a change in temperature or variation in the environment. In contrast to this type of rheology, yielding liquids (sometimes not rigorously referred to as “gels”, especially in relation to colloids) can exist in a solid-like (gel-like) state and become fluid above some defined stress and time conditions (yield stress). At low stresses, their behavior is quite similar to that of permanent solid gels, including the frequency-independent storage modulus. The gel-to-sol transition considered in colloid chemistry is treated as a case of yielding. However, in many cases, the yield stress cannot be assumed to be a physical parameter since the solid-to-liquid transition happens in time and is associated with thixotropic effects. In this review, special attention is paid to various time effects. It is also stressed that plasticity is not equivalent to flow since (irreversible) plastic deformations are determined by stress but do not continue over time. We also discuss some typical errors, difficulties, and wrong interpretations of experimental data in studies of yielding liquids.

## 1. Introduction

The term “gels” is one of the most widely used when scholars study various soft matters. The concept of “soft matter” was first proposed by deGennes when considering complex fluids and soft condensed matter [1]. These matters have two main characteristics: a complex structure and flexibility. It is intuitively clear that under this definition, a certain group of multicomponent and, most likely, multiphase materials characterized by a low elastic modulus and, possibly, but not necessarily, fluidity is considered. The rheological properties of such systems should belong to the class of viscoplastic (or yielding) media since the dispersed components of the mixture should form a certain “structure” characterized by some strength. The term “structure” does not have a strict definition either since we are talking not only about crystalline bodies but also amorphous ones characterized by some specific interaction, causing the ordering of their parts and/or orientation in space. Soft matter is widely used in production, such as tensides, polymeric fluid compounds, liquid crystals, membranes, gels, concentrated emulsions, foams and other colloids, and proteins [2].

If we are dealing with such a substance as solid “gels”, there is no doubt that they have some strength under mechanical loading. If the medium is capable of irreversible deformations, then the yield stress characterizes the strength of the internal structure. In this case, two modes of behavior of the medium can be realized: solid-like (or, more precisely, gel-like) at low enough stresses and fluid at higher stresses that exceed the characteristic threshold, the yield stress. The phenomenon of gel–sol or sol–gel transition, which is important for colloidal (but not only colloidal) systems, refers just to the transition between the two extremal cases (from a gel-like to a liquid state and vice versa).

This seemingly unambiguous interpretation of the experimental data concerning the rheology of these substances turns out to be far from being so unambiguous in practice, primarily due to the kinetic or time factor, as well as the need to operate with very low stresses, which are at the limit of the capabilities of standard experimental technique. Indeed, in many cases, it is true, and the statement that “time dependence is a rheological attribute that is not well understood” [3] is absolutely true.

We will consider mainly shear deformations, although undoubtedly, the deformation-induced transition from a solid-like to a fluid state exists for any type of stress field that is described by modern rheological models [4,5,6,7,8]. This consideration is based both on the authors’ own experimental data and on numerous results related to various soft matters that have been published in recent years.

This review has the following goals:

- To propose an unambiguous and unequivocal classification of gels and related materials based on rheological arguments;
- To summarize the results of studies of yielding liquids published during the last few years;
- To discuss some typical errors, difficulties, and wrong interpretations of experimental data in the studies of yielding liquids.

We will adhere to the following terminological definitions:

- Gels are solid bodies incapable of irreversible deformations;
- Yielding liquids are materials that can be in a solid-like state but, under some threshold conditions, can transfer to a liquid state;
- A gel-like state is the solid-like state of a yielding liquid.

## 2. Gels and Gel-like States

### 2.1. Formation of a Gel

A gel is a multicomponent system formed by a structure-forming component and an absorbed liquid, usually a low-viscosity solvent. In particular, hydrogels can be defined as highly hydrophilic three-dimensional (3D) networks composed of cross-linked natural or synthetic polymer chains capable of holding huge amounts of water (>90%) [9].

**Figure 1:**
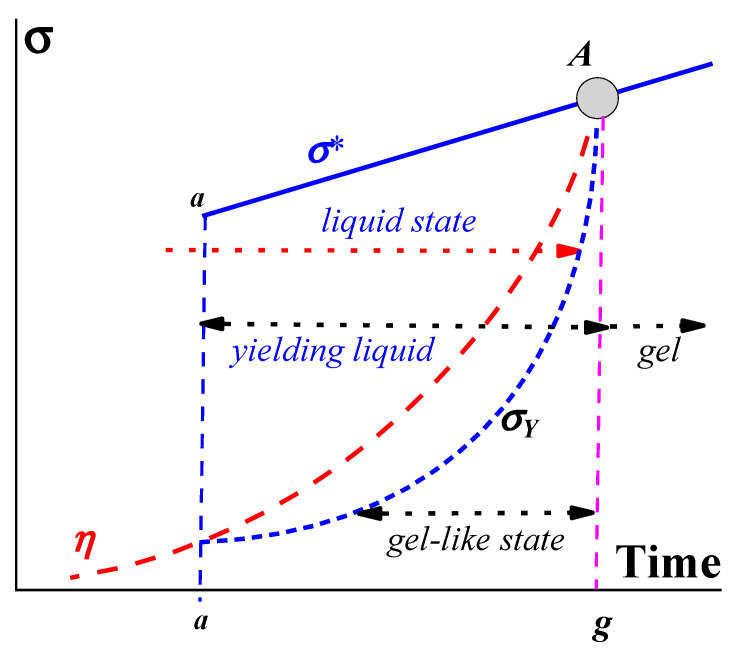
A rheological model of gelation.

The general thermodynamic understanding of such compositions is “systems with incomplete phase separation” [10]. This incompleteness is due to steric and/or kinetic reasons. Let us consider a possible rheological model of gelation (Figure 1):

The viscosity, *η*, of the initial composition of a liquid begins to grow due to its own intermolecular reactions, and at time *a* (in Figure 1), molecular interactions lead to the formation of a structure characterized by the yield stress, *σ_Y_*. This corresponds to the transition from a liquid (fluid) state to a yielding state, which exists during the time interval *a–g*. This interval consists of two parts: a gel-like state (at stresses below *σ_Y_*) and a liquid state (at stresses above *σ_Y_*), as shown by the arrows in Figure 1. The substance in a liquid state can demonstrate different rheological behaviors, including viscous flow and/or viscoelastic deformations, depending on the applied stress and time of its action. The upper limit in Figure 1 is the stress σ* (either shear or normal) at which the liquid is broken up. It should not be surprising that a liquid is characterized by a certain strength since complex liquids are always more or less elastic. Then, the destruction of such a liquid (as well as a gel), like any solid, is associated with the release of stored elastic energy. Thus, there are two characteristic parameters of the matter–macro-strength, σ*, and yield stress, *σ_Y_*. In the process of gelation, both of them increase over time and, at some point, become equal at point ***A***. This means that the object in question becomes a real non-flowing gel.

At this point, it is necessary to define the difference between gels and yielding liquids. Indeed, the term “gel” is often used by scholars in various senses. According to the definition, a gel is a non-fluid soft elastic substance with a permanent structure, and the application of external forces results in viscoelastic reversible deformations, which, when a certain critical stress state (at σ*) is reached, are destroyed. At the same time, there are many multicomponent compositions, e.g., polymer solutions with a transient structure that can exist as viscoelastic solids and become yielding fluids when an applied load exceeds some threshold. Such media (viscoplastic substances according to the standard but possibly not quite exact rheological nomenclature) are also frequently called “gels”. This extending using the concept of gels (which is not assumed in this review) is popular, especially in colloid science, where yielding colloidal substances are usually treated as gels [11,12]. Sometimes, it is also erroneously said that with an increase in the viscosity of polymer solutions and a decrease in temperature or an increase in concentration, those solutions become “gels”. But they are not gels in any way! (Certainly, it is true if an increase in concentration or a decrease in temperature does not lead to the intersection of the binodal in the phase diagram of this polymer–solvent pair).

Meanwhile, a boundary between solutions and gels may not be rigid but somewhat blurry. It depends on the nature (energy and lifetime) of the intermolecular interactions. The characteristic times of the intermolecular contacts are determined by their energy and the energy of the Brownian motion. These times can be less than the time of observation (measurements), and in this case, it is a real solution. However, if components of the dispersed phase can create long-term associates and their lifetimes lie in the range acceptable for measurements, we arrive at the transition to the temporal gel state.

True gel (or solid gel), therefore, means exclusively a non-fluid solid substance. Such a gel may transit into a fluid state only when environmental changes (temperature, pH, etc.) occur. This is true, for example, for so-called “thermoreversible gels” [13].

A substance that can be in a solid-like state and, under external forces, turn into a fluid state is called a “*yielding liquid*” (or “*yielding stress*” material). These substances (by analogy with Newtonian liquids) can also be called Binghamian liquids or media. The boundary between these two states of soft matter is determined by the *yield stress*. At stresses lower than the yield stress, such matter is considered as being in a *gel-like* or *solid-like* state, and at stresses higher than this threshold, the matter is considered to be in a *liquid state*, or *sol.* The transition between these states is traditionally (in colloid science) called a “*gel-sol*” (or vice versa) transition, although, in fact, it should be referred to as a gel-like (not gel) to sol transition.

*Gels* (solid-like gels), as a rule, are chemically cross-linked substances, the structural network of which is formed by covalent or ionic bonds. Due to the nature of these bonds, they are not able to perform the gel–sol transition under the applied external load. Yielding materials (liquids) are formed by the transient physical cross-linked network existing due to dispersed non-covalent interactions. The three-dimensional network of such a system can be reversibly (or partly irreversibly) destroyed by a high enough external load, and this is considered a gel–sol transition. This difference between the two types of rheological behavior is associated with categorizing gels into chemical gels and physical gels, where the former have permanent covalent bonds while the latter, like colloidal gels, have temporary and weak bonds [14].

The term “*plastic*” in the mechanics of solids is understood as an irreversible deformation when this deformation depends on stress only, but at a certain stress, strain does not depend on time. That is why plastic deformations are something different to flow.

The creation of a solid-like structure, within which a large amount of liquid is retained, is widespread in various technologies. Important examples of such technology include the adsorption of crude oil mixed with water and the excess water production in petroleum reservoirs [15,16,17,18], hygroscopic polymer gels [19], the development of stimuli-responsive material [19,20], 3D printing [21], medicine and food soft matters [22,23], and fiber spinning from polymer solutions. The latter case is based on the effect of the phase separation of a polymer solution jet on contact with a coagulant. The state of the system is described by Equation (1) for the Flory–Huggins parameter:

(1) $$\frac{\Delta \mu_{1}}{RT}=\ln(1-\varphi_{2})+\left(1-\frac{1}{x}\right)\varphi_{2}+\chi_{1}\varphi_{2}^{2}$$

where *R* is the gas constant, *T* is temperature, *x* is the ratio of molar volumes of polymer and solvent, and *μ*_1_ is the chemical potential (or the parameter of polymer–solvent interaction), which does not depend on *φ*_2_ and is proportional to 1/*T*.

One of the interesting and industrially used polymer gels is formed by the gel method of ultra-high molecular weight polyethylene processing into fibers, where the jet of the solution in non-polar solvent transforms to gel-like fiber under a decrease of temperature [24].

Sorbents of various types are typical examples of industrial gels; among them, hyper-cross-linked co-polymers of styrene and divinylbenzene are special gel materials. These microporous gels have a free surface of more than 1000 m^2^/g and can absorb a huge amount of liquid [25,26]. The development of new gels/sorbents continues due to the requirements of environmental safety and the oil industry, in particular. New super-absorbing cross-linked hydrogels are characterized by a swelling ratio exceeding 10 (at room temperature) [27]. Mesoporous hydrogels also belong to the group of gels with a developed surface that can be important for biomedical applications [28].

The polymer materials related to gels include weakly cross-linked rubbers containing a large number of plasticizers, as well as protein and polysaccharide gels [29,30], with their high concentration of biopolymers, which are firm enough to be self-supporting and show fractures at large deformations.

Since gels are used in such a multiplicity of applications, it is crucial to correctly characterize the rheological behavior of these soft matter, bearing in mind that their properties are mainly determined by their microstructure. Various methods are used to study gel structures in order to explain their rheology, such as small-angle neutron scattering [31], rheo-NMR [32], fluorescence measurement [33], light-scattering [34], atomistic simulation [35], etc.

The rheological analysis of soft matters consists of describing the complex characteristic properties of an object in various rheological states and features of threshold parameters–macro-strength and yield stress.

### 2.2. Rheology of Gels

Gels are materials formed at poin **A** in Figure 1 and possibly continuing to develop after point **A**. The *X*-axis may not necessarily represent time but, for example, temperature or a change in the composition of the matrix due to the addition of certain chemicals. According to the assumed definition, a gel is a solid matter without the ability to flow. The nature of the mechanical rupture of gels resembles the known mechanisms of breaking up of solids.

Structural bonds in a gel can be of different nature [36]. The limiting case is strong covalent chemical bonds between macromolecules (chemical network), and the upper limit of such gels is plasticized rubbers and plastics (such as, for example, plasticized PVC and PVC plastisols). Their rheological behavior is quite well known. In the ideal case, it is characterized by a wide rubber-like plateau on the frequency dependence of the storage modulus *G*′ (*ω*) and relatively low values of the loss modulus *G*″ (*ω*).

The characteristics of gels on breaking up, as for other solid-like materials, have many similar features. First of all, two main types of break can be distinguished: brittle rupture and elastic yielding. The latter is intrinsic for polymeric substances [37,38], although brittle rupture has also been observed for these substances [39]. Clear evidence of gel fracture was presented in [40]. It is interesting to note that the shearing point corresponding to the destruction of the gel structure was determined by critical strain rather than stress. Similar results were described in [41], where brittle fracture of protein gels was observed and fitted by using some characteristic constants.

Macro-fracture of gels (as well as other soft matters) can be observed in two modes–either as the appearance of discontinuities inside the sample due to the breaking of cohesive contact or the transition to wall slip instead of shear in volume. Direct observations of inhomogeneous deformations with a fracture zone after initial elastic strain were demonstrated for the thixotropic (“self-healing”) protein gel in [42]. Destruction can be accompanied by sliding along the solid boundary surface of the experimental cell. In this study, the effect of delayed fracture, or durability of the gel, was found. This is very similar to the stress dependence of the lifetime of solids associated with the Zhurkov–Bueche kinetic fracture model and described by the exponential law [43]

(2) $$t*=Ae^{-\gamma \sigma }$$

where t* is the time-to-break at the constant applied stress *σ*, and *A* and *γ* are parameters of the model. Gels can be viscoelastic, and, in this case, the break-up occurs after some viscoelastic (recoverable) deformation.

**Figure 2:**
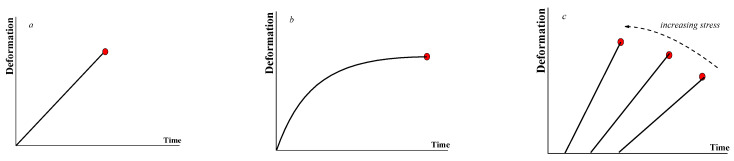
Possible modes of break-up–elastic (**a**), viscoelastic (**b**), and delayed (**c**). The last point on all curves corresponds to the time of fracture (marked by a red point).

Three possible modes, namely elastic (*a*), viscoelastic (*b*), and delayed (*c*), of break-up are shown in Figure 2.

The strength of gels in the extension was also investigated, and many publications are devoted to this method. A detailed review [44] considers various aspects of gel fracture, including its theoretical substantiation, and contains a large number of references to original publications.

To understand the physical nature of the macro-fracture of gels formed by a transient network, it is advisable to introduce some characteristic time of a determining relaxation process. Then, the possibility of break-up is determined by the ratio between the time of deformation (reversible deformation rate) and this time. This situation is analogous to the role of the Deborah Number for viscoelastic soft matters [45]. Meanwhile, two different relaxation modes can play a crucial role. The first is the characteristic lifetime of bonds or entanglements, and the second is the relaxation time of macromolecular movements. Depending on the scale of these times in terms of the strain rate, we encounter either brittle or viscoelastic fractures.

### 2.3. Yielding Materials (Liquids)

The viscoelastic properties of a matter in a gel-like state, where the network is formed by transient bonds, depend on its structure. The direct experimental proof of a solid-like behavior of the gel-like state is the independence of the storage modulus of frequency accompanied by relatively low mechanical losses. There are numerous examples [46,47,48,49,50,51,52] of such behavior, many of which are analyzed in reviews and monographs [53,54,55]. The evolution of viscoelastic properties in the transition from yielding liquid to real gel is carefully described in [56].

**Figure 3:**
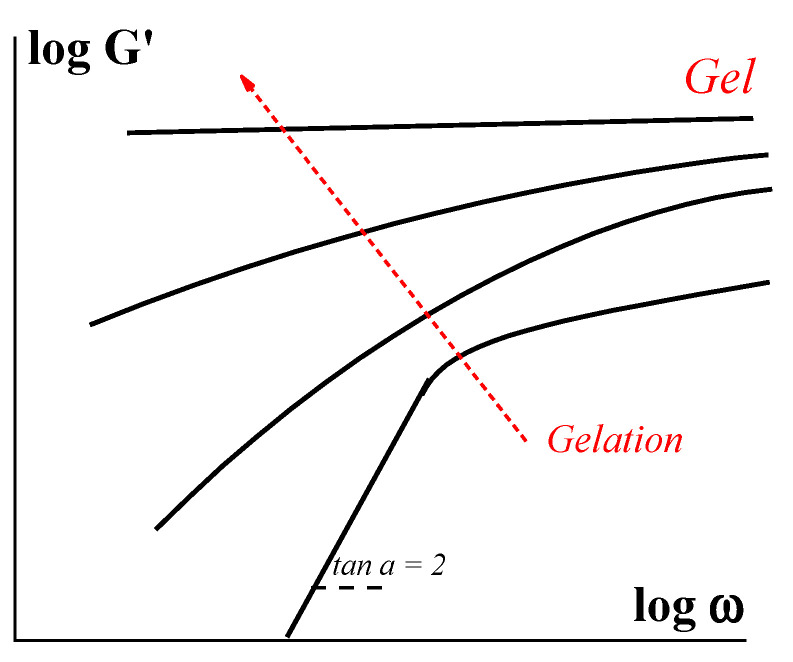
Evolution of the storage modulus in the process of gelation (scheme).

Developing viscoelasticity can be followed in parallel with the scheme in Figure 1. Initially, we deal with a usual Newtonian liquid with viscosity η_0_. Its loss modulus G″ is equal to (ωη_0_) in a whole frequency (ω) range and elasticity is negligible (G′ = 0). When a structural network appears, this is reflected in the appearance of non-Newtonian behavior, and the evolution of the storage modules occurs as schematically shown in Figure 3.

At the beginning of the process, a relaxation of the structuring liquid is close to a simple Maxwellian model, and the slope of the G′ (ω) dependence is equal to 2, which corresponds to a single relaxation time θ. Along with the development of the network, relaxation properties are characterized by a set of relaxation times that is reflected in a decrease of the slope of the G′ (ω) dependences. Finally, after reaching the gel state, we deal with a solid-like matter with the elastic modulus independent of frequency (upper straight line in Figure 3) as observed for any solid body.

**Figure 4:**
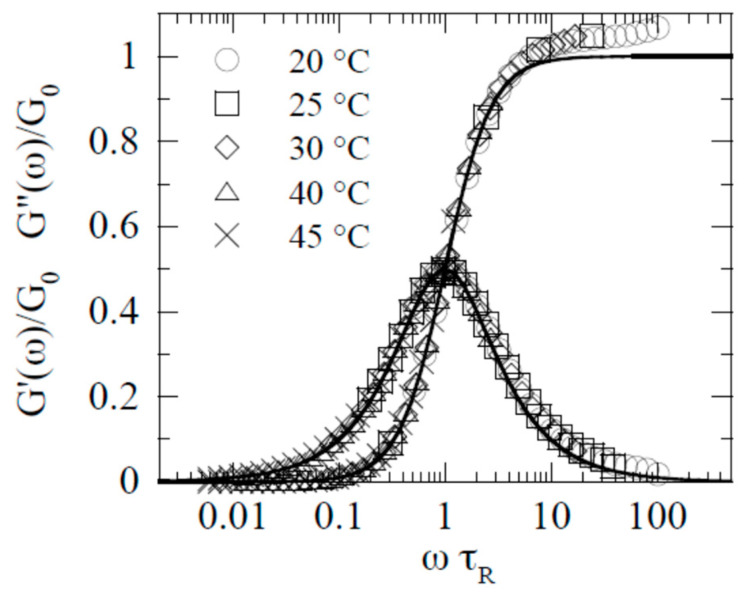
Viscoelastic properties of wormlike micelles (from [57] with permission).

The width of the relaxation time depends on the nature of the matter. An extreme case is micellar solutions, which are yielding liquids. According to the review [57], the viscoelastic properties of wormlike micelles are well-fitted by the Maxwell single-relaxation time model in a wide frequency range. This is illustrated in Figure 4, where the solid lines are built strictly according to the Maxwell equation, and *τ_R_* and *G*_0_ are relaxation time and the elastic modulus of the Maxwell model, respectively.

Such behavior in the linear region of viscoelasticity is quite typical of various micellar structures [58,59] and lamellar gels [60]. The constants characterizing the relaxation properties of such soft matters are related to the classical relationship.

(3) $$\tau_{R}=\frac{\eta_{0}}{G_{0}}$$

If a structural network is formed by macromolecular chains, the relaxation spectrum of the soft matter can be quite wide since the polymeric chains have many modes of relaxation (see, for example, [61,62]).

**Figure 5:**
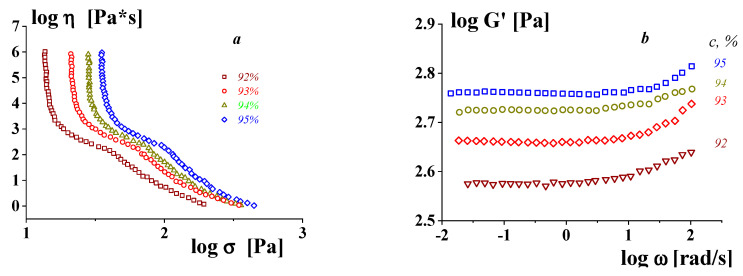
Flow curves–dependences of the apparent viscosity on shear stress (**a**) and frequency dependences of the storage modulus (**b**) in the gel-like state of low stresses for concentrated emulsions (these objects are liquid explosives.

The concept of a gel-like state relates to different substances, including those that are not usually considered to be “gels”. The viscoelastic properties of highly concentrated emulsions presented in Figure 5 are a typical example confirming this approach. One can see the correlation between the rheology of typical yielding materials (presented by the flow curves) and the frequency independence of the storage modulus in the gel-like state (at low stresses) [63].

**Figure 6:**
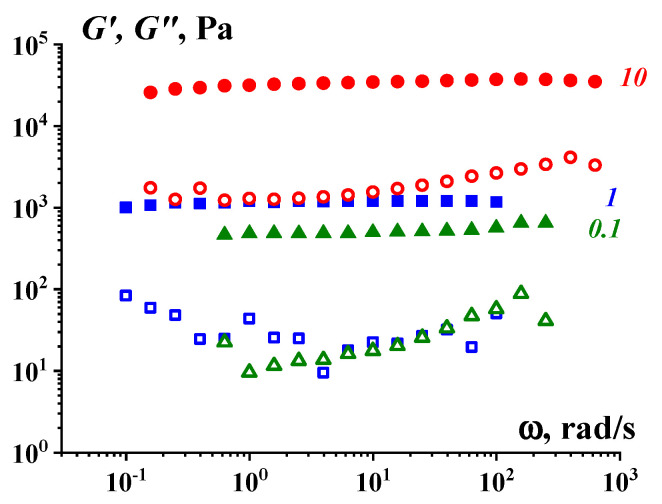
Viscoelastic properties of the aqueous solutions of poly(ethylene oxide) with different concentrations (shown in the curves) in the presence of 3 vol.% of SiO_2._ Filled symbols–storage modulus G′; open symbols–loss modulus, G″.

Figure 6 demonstrates the other typical feature of the viscoelastic properties of a gel-like state of soft matter, where an intermolecular network in the polymer solution is created by silica nanoparticles. These have much higher values of the storage modulus in comparison with the loss modulus [64].

The experimental data also show that the same ratio between the components of the complex elastic modulus is observed for the aqueous dispersion of a biopolymer complex of gelatin with polysaccharide [65]. In this material, the storage modulus is practically constant, and the loss tangent is quite small.

The above examples for different soft matters show that the gel state in yielding structured substances, as well as gels, should be considered solids, and they have the following common features: constant values of the storage modulus and low mechanical losses over a wide frequency range.

**Figure 7:**
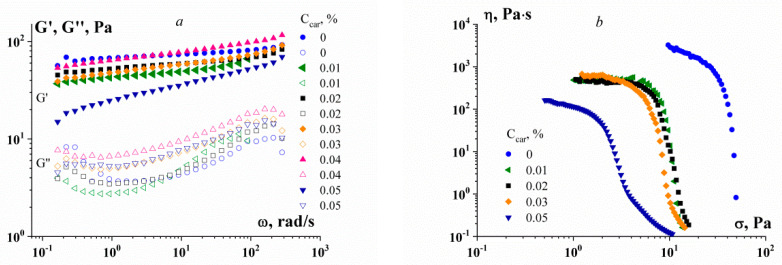
Experimental data for highly concentrated emulsions for the gel-like region (**a**) and “complete” flow curves (**b**). As an emulsion stabilizer, a mixture of gelatin (C_G_ = 0.5 wt.%) with κ-carrageenan (C_car_, wt.%—shown in the figures) is used.

Based on what has been said above, it follows that flow in the region of the gel-like state is not possible. However, there is a long tradition of “measuring” the largest Newtonian viscosity below the yield point. Historically, this is associated with the concept of a “complete” flow curve [65,66], but experimental results of this kind are also often presented in modern publications [67,68,69,70,71,72],

A rather curious example in this regard is the results of a study of the rheological properties of concentrated emulsions [47]. The object of this study was definitely yielding liquids, and this was confirmed by the frequency independence of the storage modulus (Figure 7a). However, the experimental data were also presented in the form of flow curves within the domain of the maximal Newtonian viscosity (Figure 7b). This is an obvious contradiction, and it can be assumed that the problem is related to an inadequate understanding of the experimental data at low shear rates.

**Figure 8:**
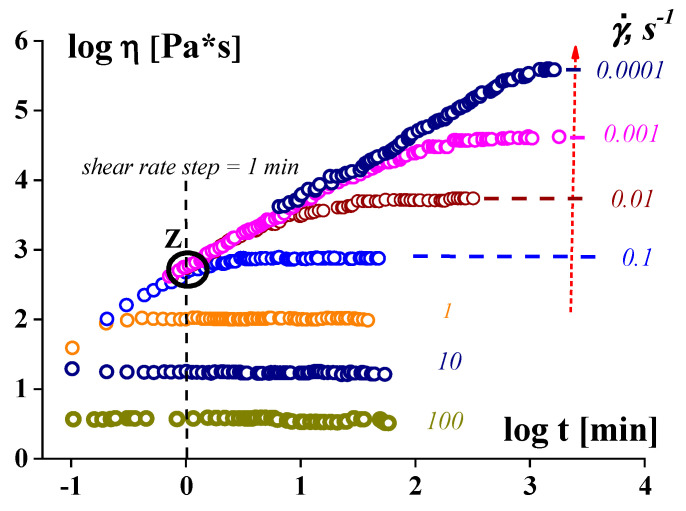
A general diagram illustrating the dependence of any false upper Newtonian limit on the time effect. The presented data were obtained for highly concentrated emulsions.

The situation is related to the peculiarities and difficulties of measurements at low shear rates, as well as at low frequencies. As a rule, measurements of the apparent viscosity depending on the shear rate are carried out by scanning the shear rate using certain steps, and the duration of the deformation at each shear rate is constant. When studying the viscous properties of yielding stress liquids, this leads to the results shown in Figure 8 (according to [73]). Here, the time step is chosen to be 1 min. At a high shear rate (exceeding 0.1 s^−1^ in this example), this time is sufficient to reach a stationary regime of the flow corresponding to the value of the real viscosity. In contrast, at slower deformations, shearing always hits point Z regardless of the specified shear rate. Then, at all shear rates over any of its range, the apparent “viscosity” seems to be the same, and this is erroneously treated as the upper Newtonian limit, not depending on the shear rate. However, if we increase the deformation time (duration of observation), we will obtain larger values of the quasi-Newtonian viscosity, as shown by the vertical arrow in Figure 8. In fact, the left envelope does not have any limit, and the apparent “viscosity” increases unlimitedly, corresponding to the approaching yield point.

The role of elasticity in transient deformation regimes during the stepwise reduction in the shear rate is also stressed in [74].

A general approach to the role of the time effect was later confirmed for various yielding liquids by measuring the increase in quasi-Newtonian viscosity while increasing the observation time [75]. This result included gel for hair dressing, foam, emulsions, Carbopol, and food products such as mayonnaise and tomato puree, which have frequently been considered as “visco-plastic”, although sometimes (contrary to this classification) many of them have been characterized by the upper Newtonian viscosity. However, it must be kept in mind that the structure in various liquids, particularly in Carbopol, may develop very slowly. Therefore, depending on the pre-formation of the sample, the experimenter can conclude whether the liquid under study is a simple one (Newtonian) or whether, due to thixotropic phenomena, it has a yield point [76,77,78]. One can find a complete analysis of Carbopol microgels as a typical example of yielding liquid in [79].

The values of the quasi-Newtonian viscosity for yielding soft matters are usually shown at the level of 10^6^–10^9^ Pa·s, and the yield stress is of the order of 10 Pa. This means that shear rates at this range of viscosity should be of the order of 10^−5^–10^−8^ s^−1^. The steady-state values of viscosity in this range of shear rates are reached in 10^5^–10^8^ s (in the interval from 30 h to 40 months). It is doubtful that anyone has actually conducted such long-term experiments, not to mention the fact that it is difficult to expect the properties of many substances to be stable for such a long time. This estimation also confirms the unreality of flow in the stress region below the yield point. All these arguments lead to the conclusion that no steady-state flow below the yield stress is possible, and the origin of apparent viscosity in yielding fluids below the yield stress is an artifact [80].

Meanwhile, none of the above denies the existence of the upper Newtonian viscosity in the rheology of multicomponent but single-phase systems such as polymer solutions. Such highly viscous solutions may visually resemble gel-like media, but they are not.

Yielding observed above the yield point and the subsequent irreversible deformation is determined by a number of microscopic structural phenomena [81], the nature of which has not been fully elucidated.

In recent years, significant progress has been made in the research of soft materials [3,82]. Modeling and theoretical studies have shown that flow arises as a result of microscopic local rearrangements involving several particles in the so-called “shear transformation zones” (in certain areas). In experiments with colloidal particles, it is possible to visualize directly the rearrangement of particles that occurs during shearing [83]. Yielding is a gradual transition, occurring to an increased extent as the deformation increases [84], which is accompanied by irreversible particle rearrangement [85].

Finally, it should be noted that yielding is a three-dimensional phenomenon [6]. Traditionally, the solid-to-liquid threshold is determined via shear stresses, although normal stresses in complex fluids can lead to new flow phenomena [86]. A quite simple method for determining the elongational yield strength from the mass of a droplet emanating from a cylindrical capillary was developed in [87]. The results of the first and second normal stress differences for yield stress liquids were reported in [86], and it was shown again that normal stress differences are quadratic functions of the shear stress.

## 3. On the Yield Stress in Soft Matter

Measuring the yield stress, σ_Y_, of viscopastic media is not such a simple task as it seems at first glance [88,89]. First of all, it is necessary to determine what is meant in a particular case by this value, i.e., an unambiguous definition of what we want to measure should be given. A milestone in this problem was the classic work of Bingham [90]. He clearly distinguished two possible states of multicomponent multiphase materials: a solid (or gel-like) state, where the material exhibits only elastic deformations, and a liquid state, where this material can flow under the action of applied stresses. The boundary between these two states is the yield stress (or yield point) σ_Y_, and its value was considered as a physical parameter of a substance.

Such an approach can often be found in modern publications on the characterization and comparison of various substances since it adequately describes the behavior of various concentrated suspensions, for example, bentonite colloidal systems [91,92], electrorheological liquids [93], and supramolecular solutions [94], as well as colloidal gels [95,96,97], gels for 3D printing [98], liquid crystal systems [99], ferrofluids [100,101], and magnetorheological fluids [102]. Yielding behavior and flow are important in many operations within the oil and gas industry [103].

A visual analysis of many model situations using the Bingham understanding of yield stress is conducted in one review [104]. Also, the Bingham model and its non-linear generalization are the basis for solving a lot of dynamic (boundary) problems. The known analytical solutions can be used in technological practice, for example, in designing the transport of cement mortars.

**Figure 9:**
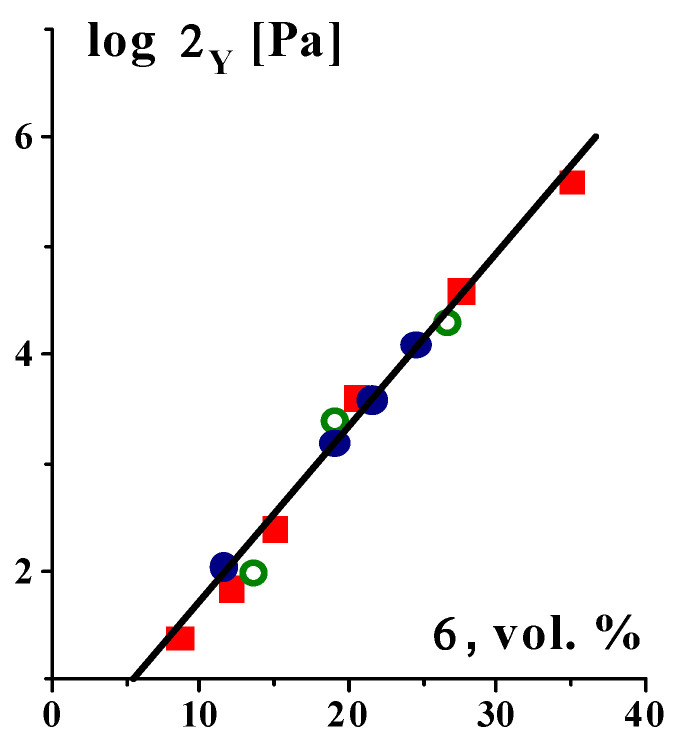
Concentration dependence of the yield stress for dispersions of carbon black (surface 300 m^2^/g) in different matrix–poly(butadiene)s with M_w_ = 1.35 × 10^5^ and 1 × 10^4^ Da and low-viscosity silicon oil–marked with various symbols; with various symbols calculated by the Casson and the Herschel–Bulkley models [106], and by G′ (σ_0_) curves. Original data.

The yield stress value reflects the strength of a structure, which is created by intermolecular interactions of various types. The upper limit of the yield stress depends both on the nature of the bonds and on the concentration of the structure-forming components. The strength of the structure in highly filled compositions can exceed 10^5^ Pa, and this is the structure formed specifically by the filler since the yield stress does not depend on the nature of the liquid matrix [105] (see the example in Figure 9).

**Figure 10:**
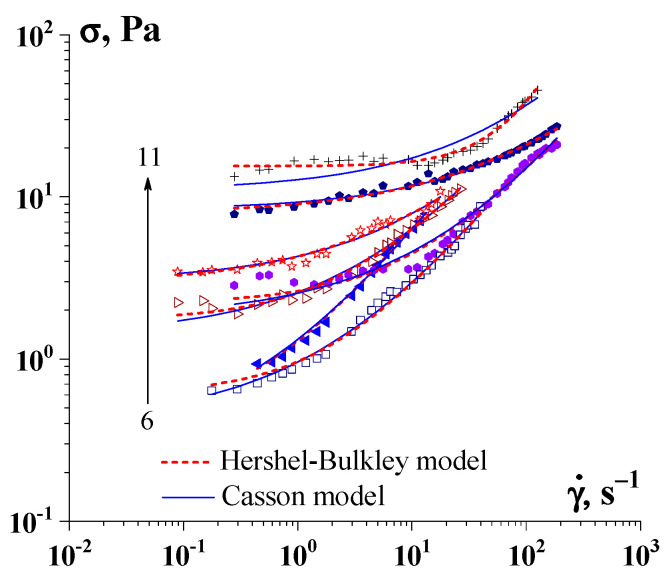
Determination of the yield stress by the Herschel–Bulkley model and the Casson model for hydrogel of gelatin-κ-carrageenan complexes (C_g_ = 1.0 wt.%). The change in the κ-carrageenan concentration is indicated by arrows, C_car_, wt.%: 6–0.100, 7–0.150, 8–0.175, 9–0.200, 10–0.400, 11–0.500.

However, this is true only for components that do not interact with each other. In fact, the matrix can strongly influence the structure formation in gels. Of course, other options are possible, in which the matrix will take part in the formation of the structure [107]. An illustrative example of the formation of a continuous structure by silica in a polymer matrix depending on the pH of the medium is presented in [108]. The formation of a three-dimensional structure has been shown in systems based on proteins with polysaccharides, the properties of which (Figure 10) are determined by the composition of biopolymer supramolecular complexes [48,66].

The measurement of large values of the yield stress presents no fundamental difficulties. However, the issue of the minimum physically reasonable values of the yield stress is of special interest. In practice, using standard experimental techniques, it is not really possible to reliably measure the values of the yield stress below 1 Pa.

However, supramolecular structures have very low strength, and in this case, the values of the yield stress may be obtained by extrapolating experimental data. Such an approach sometimes gives minimum values of the yield stress in the range of 0.01–0.1 Pa at a concentration of the structure-forming phase significantly below 0.1% [107,109,110]. Structure formation in organogels at extremely low concentrations of less than 10^–3^% has been described [111,112]. So, the level of the minimal value of the yield stress might be roughly estimated as 0.01 Pa; however, such low values of yield strength should be treated as nothing more than a value judgment.

How strong, in fact, can supramolecular structures be?

We can give the following estimation of the minimum possible strength of structure formation based on the supposition that the structure should not be destroyed by Brownian motion. This requirement is expressed in the inequality of the Péclet Number:

(4) $$Pe=\frac{\sigma_{Y}d^{3}}{kT}>>1$$

where *d* is the characteristic size of the structural element, *T* is the absolute temperature, and *k* = 1.380649 × 10^−23^ J/K is the Boltzmann constant.

This implies a possible yield limit, namely:

(5) $$\sigma_{Y}>>\frac{kT}{d^{3}}$$

For the characteristic size of the particles that form the supramolecular structure, it is reasonable to take the typical size of colloidal particles or the wavelength of visible light (0.4 µm). Hence, it follows that the minimum possible strength of the structure in yielding media can be no lower than 0.01 Pa, which roughly corresponds to the minimum values of the yield strength obtained by extrapolating the experimental data.

Currently, we are far away from the historical understanding of the yield strength described in the pioneering work of Bingham. A modern understanding of the concept of yielding was discussed in several recent publications [113,114,115].

The experimental measurement of the yield stress, σ_Y_, should be based on a rigorous definition of this quantity. Indeed, after the question of whether there is a yield point, a counter-question follows: What is it? (that is, first of all, a definition of what the questioner understands by this value should be given). The concept advanced by Bingham seems quite clear [90]. However, there are some theoretical and practical problems in determining the yield stress, even if we remain in the Bingham paradigm. Indeed, the yield point is defined by some equation (whether it be the Bingham, Herschel–Bulkley, Casson, or any other fitting equation; see Figure 10) and extrapolation of the experimental data to the limit at $\dot{\gamma }$ → 0.

**Figure 11:**
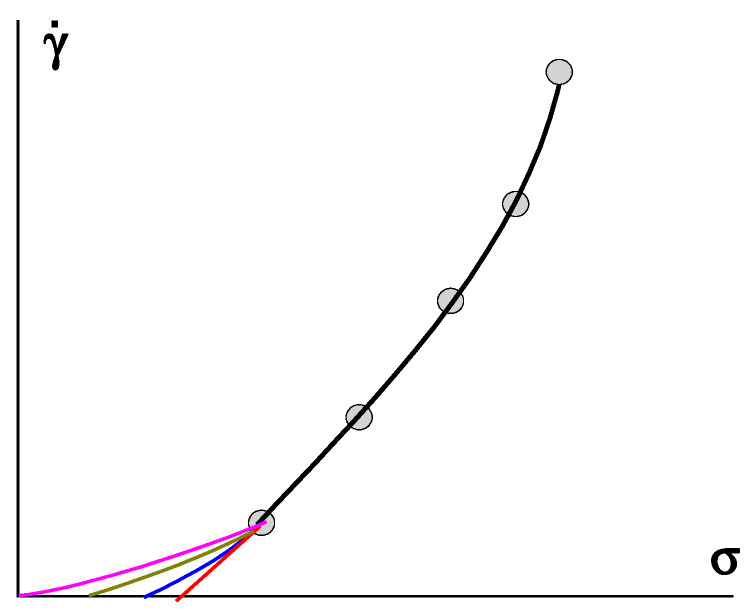
Various methods and results of extrapolation (number of lower curves) of experimental data–points and the fitting curve.

However, any extrapolation procedure leaves room for doubt, as illustrated in Figure 11, where different possible ways of extrapolating are shown. This procedure is especially dangerous when using a wide range of shear rates and presenting the experimental data in a log scale. It can be seen that it is possible to come to very different values of the yield stress (points of intersection of the lower curves with the σ-axis at $\dot{\gamma }$ = 0) and even the possible of the absence of a gel-like domain (*σ_Y_* = 0).

**Figure 12:**
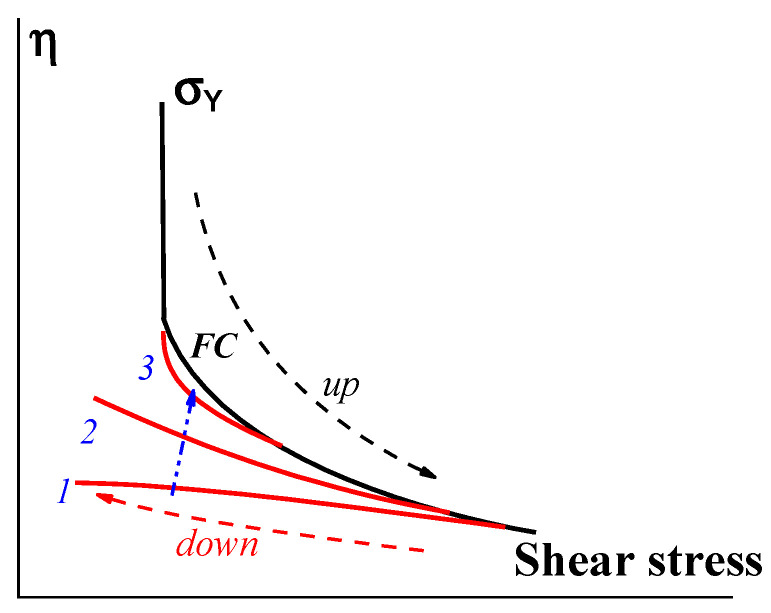
Stress up-and-down scanning in measuring the flow curve. Curves 1, 2, and 3 correspond to a decreasing rate of scanning in decreasing shear stress (along the blue arrow).

Bingham’s yield stress is a limiting case that may be true for compositions in which the dispersed component forms a rigid structure instantly breaking down at a certain stress, σ. However, even in these cases, the reverse restoration process can continue for a long time, and the different rates of approach to the initial yield stress (depending on the scanning speed) indicate the thixotropic nature of the substance (Figure 12).

**Figure 13:**
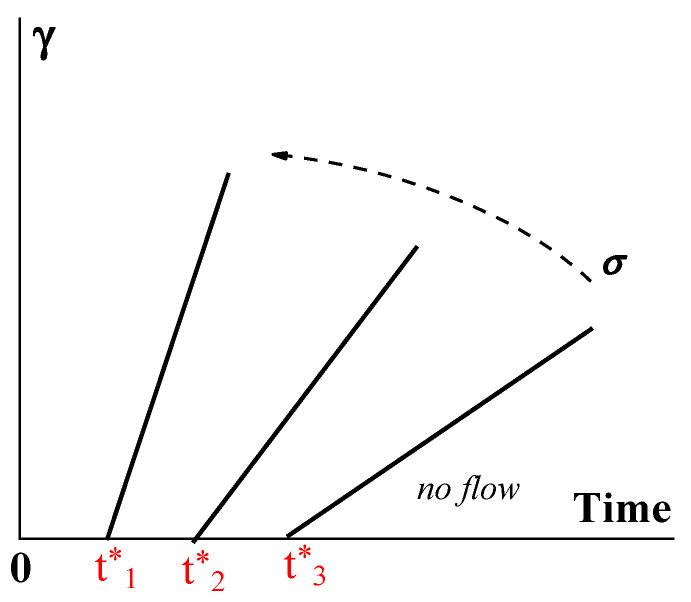
Developing deformation γ on time–Bingham yielding. The dotted arrows show the increase in shear stress.

The gel-like structure can be different due to its physical origin, and consequently, its breaking up can happen in different ways. For example, instead of Bingham’s fragile jump-like transition from the gel-like to the liquid state, this transition can happen over time [116,117]. Then, the structure can be characterized by the lifetime t* decreasing (from t*_3_ to t*_1_) along with the increase in stress. Therefore, the flow does not begin immediately after the application of the load but only after some time, which characterizes the durability, or lifetime, of the structure (Figure 13).

**Figure 14:**
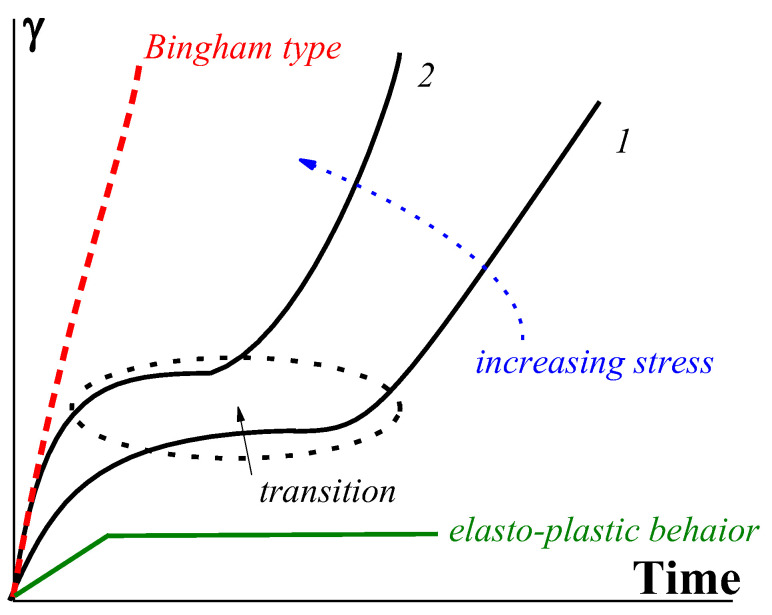
Development of deformations in transition from the gel-like to plastic state (the stress for curve 2 is higher than that for curve 1).

The function t* (*σ*) reflects the strength of a gel-like structure and thus replaces Bingham’s yield stress, *σ_Y_*. The slope of the straight lines in Figure 14 corresponds to the viscosity of a matter in a liquid state (above the gel-like-to-liquid transition).

The durability of rigid structures (in concentrated suspensions) is characterized by a rather strong dependence on stress. For example, the exponential-type t* (*σ*) dependence in yielding materials was proposed in [118]:

(6) $$t*=Be^{-\beta \sigma }$$

where *B* and *β* are empirical constants in this equation. This equation is quite similar to Equation (1), fitting the strength of a material overall.

The time effects create a serious principal difficulty in finding the “true” yield point (which in reality may not exist), and the time effect leads to the conclusion that the yield stress should not be considered a material property since it depends on the prehistory of deformation and the rest of the sample [119]. Time-dependent effects were carefully discussed in [117], with the evident conclusion that the time evolution in aging and shear rejuvenation should be the basis for estimating the flow behavior of thixotropic substances. 

The rheological properties of yielding materials are similar to those of non-fluid gels at $\sigma <\sigma_{Y}.$, where only small elastic deformations are possible. At $\sigma >\sigma_{Y}$, these elastic deformations are negligible in comparison to the irreversible deformation of flow, and therefore, they are not shown in Figure 13. However, it is reasonable to assume that the deformation during the initial stage of loading at *σ* > *σ_Y_* is not elastic but viscoelastic. Then, the development of deformations at a certain *σ* = *const* (in measuring compliance as a function of time) takes place as shown in Figure 14 (as described, for example, for various materials in [118,120,121,122,123]).

At sufficiently low stresses, the time dependence of deformation comes to a plateau, which becomes shorter and higher as the applied stress increases. Then, a very sharp increase in deformation happens, reflecting the shear-induced solid-to-liquid transition (as indicated by the oval in Figure 14) to stationary flow. The slope of the last sections of the curves (shear rates) obviously increases as the stress increases. The appearance of a plateau is characteristic of the elasto-plastic type of mechanical behavior.

**Figure 15:**
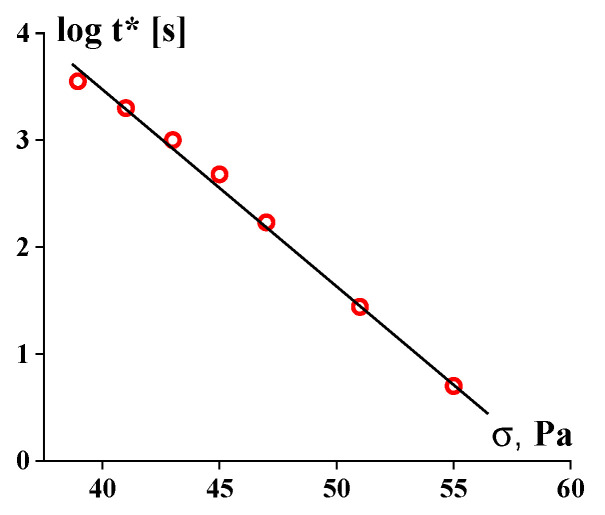
Long-term shear-induced solid-to-liquid transition for 51% suspension of Kaolin.

The dependences of such a type presented in [120] can be reconstructed to give the t* (σ) dependence, which reflects the durability of a solid-like structure. This is shown in Figure 15 (based on the data from Figure 4 in this publication).

These experimental data are approximated by the standard exponential dependence (5) with β = 0.414 Pa^−1^.

Therefore, two limiting types of rheological behavior should be distinguished: Bingham’s one with an immediate increase in deformation at $\sigma >\sigma_{Y}$ and viscoelastic behavior at *σ* < *σ_Y_* (for soft matter). The shear-induced transition from initial viscoelastic deformations to flow might not be sharp, as shown in Figure 14, but smooth. The delayed break-up (or durability) of a structure in the gel-like state is superimposed on the viscoelastic behavior of yielding materials.

The gelled crude oil is one of the most interesting substances, demonstrating a wide range of rheological effects intrinsic to yielding materials. Different time-dependent effects are observed for these objects [124]. The structure of these objects is formed by crystallizing paraffin and other components of crude oil, and this structure is sensitive to the time, temperature, and shearing history of the sample.

The main rheological feature of waxy oils is their thixotropy, directly linked to the gel-like-to-fluid transition [125]. This type of rheological behavior is analytically described by various models [4,5,121,126]. The commonly accepted approach to modeling reversible structural processes induced by shearing is based on the introduction of a certain conditional factor *β* for the notion of “degree of structuring”, which cannot be rigorously defined. This factor depends on the shear rate. The kinetic equation describing the evolution of *β* can be proposed in the following rather general form [127]:

(7) $$\frac{d\beta }{dt}=k_{1}{\left(1-\beta \right)}^{n}{\dot{\gamma }}^{\alpha }+k_{2}\beta^{m}{\dot{\gamma }}^{\nu }$$

where all constants in this equation are empirical parameters.

More common kinetic equations of the same type and their tensorial generalization were proposed in [128].

Equations of this type reflect both kinetic processes–the forward and reverse structure evolution and the role of shearing in both. Meanwhile, the process of structuring can continue for a long time in a gel-like structure at rest [129]. Such “physical aging” is typical of biological objects [130,131], including systems containing proteins and polysaccharides [132,133], and is accompanied by conformational changes in macromolecules and the microstructure of gel-like systems over time.

These structure transformations occurring over time (thixotropy) should not be confused with viscoelastic deformation [134], which is also associated with time effects [135], although the timescales of both processes can be superimposed.

The recovery of the initial structure disturbed by shearing can only be partial [136,137], and this requires an improvement of the model, taking into account these effects [138,139]. This means that the yield stress for thixotropic soft matters can be different depending on the prehistory of deformations reflecting a different structure of the matter in the gel-like state. These properties of thixotropic media are inherent not only in gelled waxy oil but also in other multicomponent materials, for example, Laponite suspensions [140], hydrogels of polysaccharides [141,142], colloidal gels [143], Carbopol [77,78], and as-spun fibers from polymer solutions.

One of the consequences of the time effect is the bifurcation of the flow curve of a thixotropic liquid, which is usually associated with its yielding [144]. This is the reason for the shear banding effect. It consists of the movement of a sample separated into two layers with different rheological properties (or even a motionless layer near a wall of the measuring device or an industrial unit and the flowing layer) [5,145,146,147]. Such rheological effects are undoubtedly associated with the evolution in local particle rearrangements, leading to such localization phenomena as shear banding and bifurcations [148,149].

Using oscillation experiments over a wide frequency range enables quantification of the time effects of yielding shown in a general form in Figure 1. This approach was (and remains) quite popular in searching for the correlation between the evolution of the dynamic modulus and direct observations, for instance, of paraffin crystallization in crude oil [150,151,152] and the structure formation of proteins in the aqueous phase [153].

**Figure 16:**
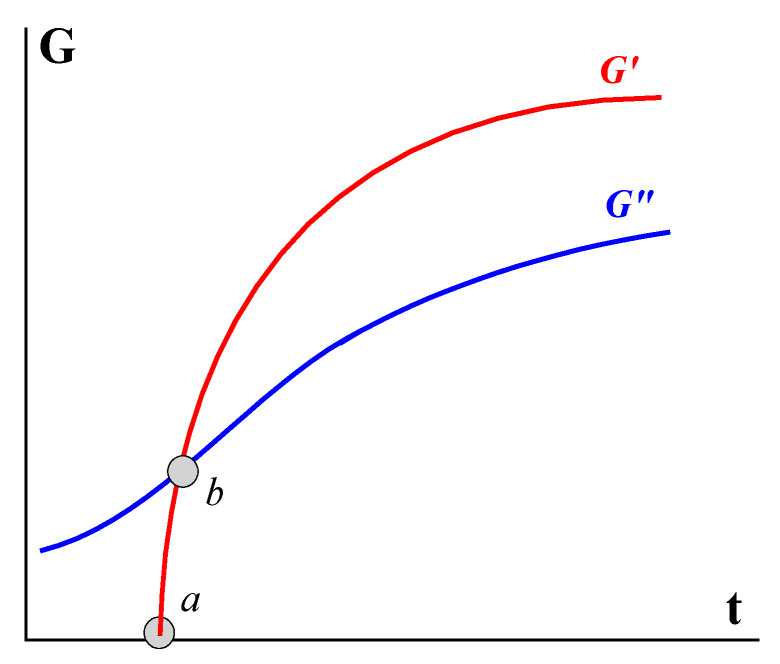
Evolution of the storage G′ and loss G″ moduli in isothermal gelation at ω = const.

The typical evolution of the components of the elastic modulus over time, *t* (at a certain frequency), for various yielding materials is exhibited in Figure 16. Point *a* can be conditionally considered the beginning of the process, and point *b* is the moment of gelation, but it is evident that the gelation occurs over time and approaching the final state is rather long. The timescale can be minutes or even days. In addition, the position of the *G*′ (*t*) and *G*″ (*t*) curves shifts depending on the fixed frequency, and the frequency dependence of the position of point *a* (or *b*) can be used as a quantitative characteristic of the kinetics of gelation.

**Figure 17:**
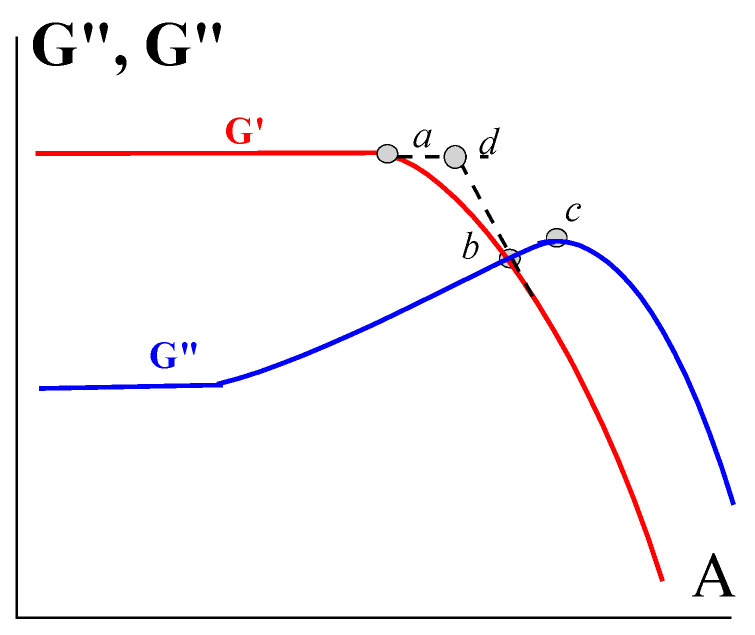
Amplitude dependences of the storage *G*′ (A) and loss *G*″ (A) components of the complex dynamic modulus showing the transition to the non-linear viscoelastic behavior at constant frequency.

The method of characterization of the yield stress based on measuring the amplitude dependences of the storage and loss components, G′ (A) and G″ (A), respectively, at some constant frequency, ω = *const*, has become quite popular. A scheme of such measurements is shown in Figure 17.

These so-called “large-amplitude-oscillatory-shear” (*LAOS*) measurements show the existence of the linear domain of viscoelastic behavior for the solid-like state of gels (where the components of the elastic modulus do not depend on the amplitude of shear) and the transition to the non-linear domain (where they depend on the amplitude). These experiments are referred to as “large-amplitude”, although their sense is not in the “large” absolute value of the amplitude used but as a marker of the linear-to-non-linear transition. For rigid gels, this transition can happen at amplitudes of less than 0.1%, although other materials continue to remain linear at deformations exceeding several hundred percent. The point of this transition is most likely related to the critical linear strain that is valid for at least some soft matters [122].

The linear-to-non-linear transition corresponds to uncertain structure transformations, and a natural suspicion arises that this effect is associated with yield stress. So, it is reasonable to try searching for correlations between the yield stress observed in shearing and characteristic points at curves in Figure 17. One can note a few characteristic points in this figure. This is point *a* corresponding to the deviation from the linearity of the *G*′(*A*) dependence (although another method for estimating this point can be proposed). Then, there is the crossover point *b*, where $G^{\prime }=G^{\prime \prime }$ showing the transition from the prevailing elastic to dominating plastic behavior. The point *c* at the maximum of the *G*″ (*A*).

These points correspond to the characteristic stresses *σ_a_* = *G*′*_A,__a_* and *σ_b_* = *G*′*_A,b_* where the subscripts *a* and *b* correspond to the values of the modulus and the amplitude at points *a* and *b*, respectively. Now, it is necessary to answer a question:

Does a correlation between *σ_Y_* found in the shearing experiment by the extrapolation procedure, on the one hand, and *σ_a_* (or *σ_b_*), on the other, exist?

Experiments conducted with a variety of soft matters (polysaccharide–protein hydrocolloids, highly concentrated oil emulsions, Kaolin or SiO_2_ suspensions with different concentrations of the solid phase, Mayonnaise, and so on) showed that the *σ_Y_/σ_b_* ratio for the studied subjects differs, varying from 1 to 10 [154,155,156]. Moreover, this ratio depends on the frequency at which the modulus is measured. So, it is rather difficult to use the LAOS method for the estimation of *σ_Y_*, especially bearing in mind that *σ_b_* depends on frequency. Careful analysis of LAOS data (for Carbopol solution) showed that *σ_Y_* ≅ *σ_d_* (for a single frequency), while for other characteristic points in Figure 17, the ratio of σ_Y_ to the stress at different points varied in the range of two to ten times [155]. It was also found that the loss tangent at the maximum on the amplitude dependence tan δ_max_ is a rather interesting value since it is proportional to the characteristic reciprocal frequency, ω^−1^. If we assume that the position of δ_max_ characterizes the yielding point, then it appears possible to obtain the time sweep of yielding or dynamic yield stress [155]. It would be interesting to extend this approach to other soft matter in order to confirm the generalizability of this conclusion.

The kinetic factor in the structure of soft matters and consequently their yield stress, as well as the whole complex of the rheological properties, depend not only on the prehistory of deformations but also on the thermal history, which influences the kinetics of the structure formation in the transient network of secondary bonds [157,158,159], crystallization in the case of waxy oils [123]. So, it is necessary to consider these materials as thermo-kinematic sensitive substances [160,161].

Moreover, as discussed above, various materials in the gel-like state may exhibit viscoelastic rather than purely elastic behavior [4,5,138,162,163]. This should also be included in a complete visco-elasto-fluid thixotropic rheological model of real yielding stress liquid [164,165,166]. This complicated model has been examined, although there are difficulties in determining the constants of the model [128,160,167]. Such a detailed description of the rheological properties of process fluids, including the measurement of a large number of empirical constants, may seem excessive. However, this is not just a matter of being curious. It is dictated by real technological problems associated with the pipeline transportation of such media as crude waxy oil. The increase in yield stress during forced or planned shutdowns of the pipeline is of crucial technical and financial importance for the restart [168,169,170,171]. Numerical simulations of the flow of yielding fluid [172,173] have provided a better understanding of the fundamental properties of yield stress fluids in many applications relevant to natural and engineering sciences.

The complex nature of the rheological behavior of real fluids forces us to approach with caution the simplified methods of estimating the properties accepted in technological practice. The results of “simple” evaluations may give incorrect estimates of the expected behavior of the material and not coincide with the characteristics of the fluidity obtained with more rigorous experiments [174].

## 4. Sol–Gel Transition

The sol–gel transition is represented by the diagram in Figure 1. This already suggests that such a transition occurs over time, although it can also be considered as a transition through the yield point since, according to the physical meaning, this is a transition from a liquid state to a solid-like state.

As emphasized above, it is necessary to distinguish between a sol-to-gel transition to a solid gel state and a sol-to-gel-like transition to a yielding material (liquid), although the term “gelation” is used for both processes.

**Figure 18:**
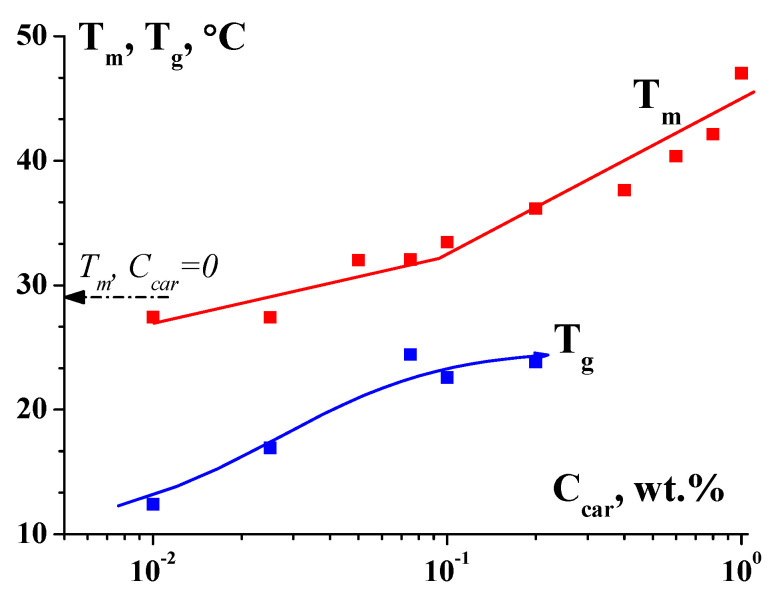
Dependence of melting (T_m_) and gelling (T_g_) temperatures on the κ-carrageenan-to-gelatin mass ratio Z in mixed hydrogels; gelatin concentration C_G_ = 1.0 wt.%. Original data.

Traditionally, people working with weak organic and especially bioorganic compounds prefer to talk about sol–gel (or gel–sol) transitions, while those who work with coarser multicomponent materials interpret them as yielding. Meanwhile, in both cases, the physical content is the same—the formation of a network of the transient bonds of various types in a liquid. Moreover, the situation is quite common when the same composition, depending on the temperature, can form a true gel (below the glass transition temperature, T_g_). And at higher temperatures (above the melting temperature, T_m_), the composition becomes viscous liquid. In the temperature range T_g_–T_m_, this is a yielding material (Figure 18) [66].

One can find a lot of pictures showing a gel-like structure in multicomponent compositions (e.g., [153]).

It is quite evident that gelation leads to the transformation of the viscoelastic properties of the matter. This basic concept was developed based on experiments with stoichiometric balanced cross-linking PDMS [175,176]. It was shown that the gel point is characterized by the equality of G′ (ω) = G″ (ω) over a very wide frequency range (but not at a single point, as assumed sometimes). In this threshold state, the following simple law holds:

(8) $$G^{\prime }\left(\omega \right)=G^{\prime \prime }\left(\omega \right)=K\omega^{1/2},$$

where *K* is a temperature-sensitive factor.

**Figure 19:**
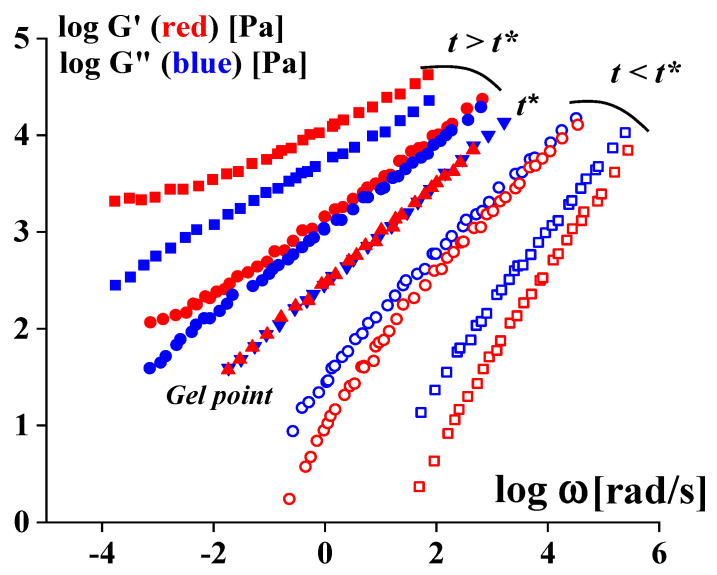
Experimental data illustrating an evolution of viscoelastic properties at the liquid-to-gel transition, according to a concept formulated in [176]. *G*′ (*ω*) and *G*″ (*ω*) are used in the reduced form, and the constants are omitted; every pair of curves corresponds to the different moments of the gelation.

Figure 19 shows how viscoelastic properties of the liquid before gelation (at *t* < t*) pass into properties of a typical gel (at *t* > t*), and this is accompanied by a change in the ratio of *G*′ and *G*″: in a liquid state, *G*″ > *G*′, and in a gel (or gel-like) state, *G*″ < *G*′.

In addition, it was shown that at the gel point, a very simple relationship exists for the relaxation modulus, *G*_r_ = *S·t^−^*^1/2^, and only a single parameter *S*, referred to as the “strength” of a gel network, is enough to characterize the gel properties. The development of this approach for stoichiometric imbalanced cross-linking was discussed in [177]. Initially, this approach to determining the gel point was developed for chemical gels. Later, it was shown that the same method can be applied to sol–gel transition for gels formed by transient bonds [178]. One can find some examples of the application of this approach in [179,180,181].

The sol–gel transition is a rather special state of the system associated with the formation of the space percolation network. In the publications of Winter et al. cited above, as well as in the review of earlier studies [182], it was shown that the rheological behavior in the vicinity of this critical point is characterized by power law dependences of viscoelastic functions on frequency or time. These power-law dependences were treated as a consequence of the fractal structure of the matter near the gel point [183]. It was also shown that the approach to the gel point is accompanied by the formation of space clusters in the pre-gel state [184]. Typically, in quiescent conditions, attractive colloids in low-concentrated solutions form fractal gels structured into two length scales: the colloidal and the fractal cluster scales. Two models that account for the structure and the rheological properties of such colloidal gels were derived from [185].

Both the agglomeration and the deagglomeration (breakdown) of the particle network in polymer nanocomposites are affected by the shear flow, resulting in shear-induced liquid-solid (sol–gel) transition and shear-induced solid-liquid (gel–sol) transition, respectively. It was shown [186] that the percolation threshold of both transitions under shear-induced agglomeration and breakdown processes depends on the shear rate under a steady shear condition. A scaling relation is suggested to describe the percolation threshold at a low shear rate. The critical strains at both sol–gel and gel–sol transitions are determined by the distance of the particle concentration to the percolation concentration.

The most characteristic feature of the rheology at the gel point is the independence of the loss tangent on frequency and/or time [175]. The scaling of the rheological function in the vicinity of the gel point for physical gels was observed and discussed in many publications (e.g., [187,188]). This issue was carefully discussed for both colloidal clay suspension and polymer solutions in [189]. This study not only confirmed the universality of the scaling concept for gelation near the gel point for various objects but also showed that exponents are the same: the gelation either occurs over time or due to temperature scanning in the non-isothermal mode.

This approach proposes a fundamental understanding of the evolution of the relaxation properties at the sol–gel transition. However, the application of this approach in practice requires cessation of the gelation process at different moments and measuring the viscoelastic properties of a set of samples obtained at these moments. Therefore, it is impossible to determine the gel point during the ongoing kinetic process since a single point at which *G*′ = *G*″ (at some frequency) obviously does not correspond to the gel point.

In research and technological practice, the sol–gel transition is considered not only as the formation of a real non-fluid gel but also as the appearance of a yield point. In addition, this transition is frequently considered not as an isothermal process but rather as a temperature transition, something like “melt temperature” [190,191]. However, in all approaches, the sol–gel transition is a kinetic process, which can take a long time. Therefore, the temperature transition can be extended along the temperature scale with a gradual transformation of the structure.

Typical results regarding the biopolymer (gelatin) aqueous solution were as follows [153]. The time evolution of the modulus continued for rather a long time in the range of the supposed sol–gel transition (24–28 °C), and the temperature strongly influenced the rate of gelation.

The authors [192] took the crossover point as the moment of sol–gel transition at the lowest frequency used, where *G*′ and *G*″ were proportional to ω^2^ and ω, respectively, although the experimental data showing the dependence of this moment on the frequency were also presented. This is a quite common technique for determining gel time at various temperatures [66,192,193,194,195], although (as stated above) this method does not correspond to the real transition and should be considered conditional for usage in technological applications.

**Figure 20:**
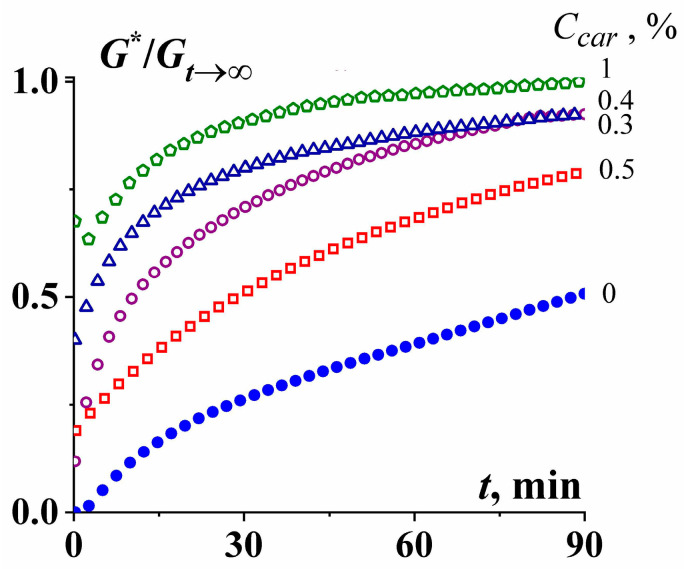
Kinetics of gelation as followed by an increase of the dynamic modulus G* normalized by its limiting value, G*_t→∞_, at C_G_ = 2 g/100 g and different concentrations of κ-carrageenan.

Figure 20 demonstrates that the addition of κ-carrageenan to gelatin solution increases the rate of gelation in the isothermal conditions as followed by changes in the time dependence of the complex elastic modulus, G* (*t*), for samples with different κ-carrageenan content [153].

The sol–gel transition of diluted colloidal dispersions is so barely noticeable that special highly sensitive detection methods are required [196].

The temperature and frequency dependences of rheological parameters can sometimes be considered interchangeable. This is an attempt to apply a similar method to the temperature-frequency equivalence in the linear viscoelasticity of polymers. In the case of viscoelasticity, this principle only works if the shape of the relaxation spectrum (as a reflection of the internal structure of the substance) does not change. In the case of gelation, both an increase in temperature and a reduction in the frequency helps to accelerate the gel-to-sol transition (“melting” of the gel). However, the internal transformation of gels characterized by rheological methods may not be similar to the temperature-induced transition. Therefore, the influence of these factors may be qualitatively different, so frequency–temperature superposition during gel melting or gelation is hardly possible.

Moreover, the gelation process can include stages of different types. In this case, using multiwave analysis can provide richer information than traditional single-frequency measurements of the elastic modulus [197]. Simultaneous determination of the complex modulus at different frequencies can be implemented using a non-harmonic input signal with further decomposition of the input and output signals using the Fourier transform method [198].

Of course, the most rigorous method for establishing the sol-to-gel transition point is to measure the evolution of the elastic modulus at different frequencies accompanied by an in-depth analysis of the relaxation spectrum at different stages of the process. However, in practice, we need simpler and faster methods, especially if we are interested in the outcomes of comparing different compositions. So a single-frequency technique (at a low frequency) can be useful, although the gelation point should not be determined by the crossover point on the *G*′ (*t*), *G*″ (*t*) dependencies but approximation of the *G*′ (*t*) dependence to the point that conditionally corresponds to the approach to the limiting plateau value. However, this approach may not be completely reliable either since many gelling systems “live” for a rather long time due to the slow transformation of the internal structure.

**Figure 21:**
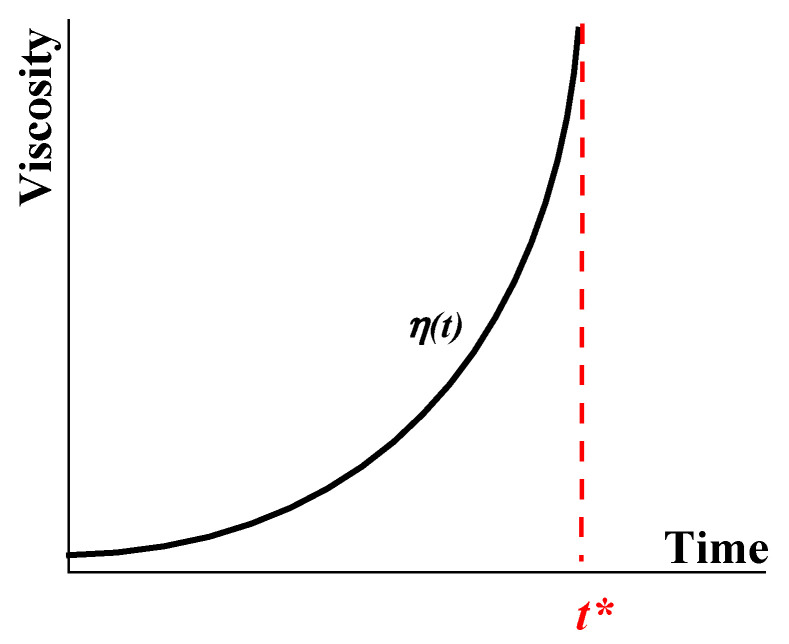
Typical evolution of viscosity over tine in the gelation process up to the gel point t*.

In technological practice, quite simple “visual” methods of the sol-to-gel transition are widely used. They are based on the standardized estimation of the moment of the loss of fluidity. Some simple proposed methods for finding the yield transition are based on elongation deformation considering the drop formation during the extrusion of a yield stress fluid in the air [87,199,200]. Observation of the “falling ball”, displacement of the meniscus, the capillary method [201,202], the inclined plate method [203], and determination of the cloud point of crude oils, or the temperature of wax appearance (WAT), according to ASTM D-5853, do not provide the necessary accuracy and reliability in measuring the gelation time, although they can be useful for preliminary characterization of the samples. Another simplified method for estimating the sol–gel point can be used based on measurements of the time dependence of the apparent viscosity at constant shear stress. This dependence is shown in Figure 21.

The analysis of the *η*(*t*) curve can be based on different analytical approximations, including a simple exponential formula:

(9) $$\eta (t)=\eta_{o}e^{\alpha t}$$

where *η_o_* is the initial viscosity of the substance in the sol state, and *α* is an empirical parameter.

It can easily be seen that this equation does not allow for determining the limit η→0 and, therefore, does not satisfy the criterion of the sol–gel transition. However, in many cases, this equation (or equations of this type) fits well with experimental data over a wide timescale. Then, the gel point is conditionally assumed as the time when the viscosity reaches a definite limit, for example, 10^3^ Pa·s. This is a rather high viscosity close to the loss of fluidity. A quite similar criterion for curing is frequently assumed in the technology of thermo-setting resins.

This review does not address the problem of the relationship between the rheological properties of gels and their application characteristics. However, such a connection undoubtedly exists. An interesting example in this regard is the observation of adhesive characteristics of hydrogels in biological objects [204,205]. Correlation between adhesive and rheological properties (especially in the range of large deformations) will lead to optimization of the required material strength.

## 5. Conclusions

It is necessary to distinguish solid gels and yielding liquids, which can form a solid-like (gel-like) structure that is destroyed by increasing stress. The proposed rigorous definition of gels as solid materials contradicts the habitual treating of colloidal yielding substances as “gels” but advances a general understanding of the rheological behavior of all materials demonstrating yield stress (not correctly called viscoplastic media).

The main peculiarity of various soft multicomponent subjects is the ability to transit from a solid-like to a liquid state due to incomplete phase decomposition. This leads to the formation of a structured network with some strength, in which a significant amount of solvent is immobilized. The transition between these states—also sometimes called the “gel-sol transition”—can be caused by external thermodynamic factors, which are typical for solid-like gels (temperature, changes in the environment, etc.) but can also occur under the action of applied mechanical forces. It is the latter case that is inherent in a large number of so-called “yielding materials” (liquids), and the solid-to-liquid transition takes place at some threshold, certified as yield stress. This point is quite clearly determined for rigid and fragile structure networks, but in the vast majority of real materials (such as lubricants, paints, foodstuffs, clay, soils, biological hydrogels, and natural phenomena such as mudflows), this transition can be extended over time. The time effects of such objects are similar to the durability of a solid-like structure and its deformation-induced thixotropy. This leads to practical uncertainty in estimating the solid-to-liquid transition point.

Gels and yielding materials occupy a huge and expanding place in our lives and in modern technologies. New materials of this type are constantly being created. At the same time, rheology remains one of the main methods for assessing the properties of any materials. This raises two obvious problems. The first of these consists of the study and establishment of correlations between the rheological properties and the structure of gels, including interaction between dispersed particles and interfacial interaction. The second task is to develop technological methods for evaluating the rheological properties of gels that would adequately reflect their applied properties.

## Data Availability

The data that support the findings of this study are available from the corresponding author upon reasonable request.
